# Optimal cardiovascular health is associated with slower cognitive decline

**DOI:** 10.1111/ene.16139

**Published:** 2023-11-28

**Authors:** Naomi Vidal Ferreira, Natalia Gomes Gonçalves, Claudia Szlejf, Alessandra C. Goulart, Itamar de Souza Santos, Bruce B. Duncan, Maria Inês Schmidt, Sandhi Maria Barreto, Paulo Caramelli, Natan Feter, Raphael Machado Castilhos, Luciano F. Drager, Paulo Lotufo, Isabela Benseñor, Claudia Kimie Suemoto

**Affiliations:** ^1^ Center for Clinical and Epidemiological Research, Hospital Universitario Universidade de Sao Paulo Sao Paulo Brazil; ^2^ Faculdade Adventista da Amazonia Benevides Brazil; ^3^ Division of Geriatrics, Faculdade de Medicina Universidade de Sao Paulo Sao Paulo Brazil; ^4^ Department of Internal Medicine, Faculdade de Medicina Universidade de Sao Paulo Sao Paulo Brazil; ^5^ Postgraduate Program in Epidemiology Universidade Federal do Rio Grande do Sul Porto Alegre Brazil; ^6^ Hospital de Clínicas de Porto Alegre Universidade Federal do Rio Grande do Sul Porto Alegre Brazil; ^7^ Deparment of Preventive and Social Medicine, Faculdade de Medicina Universidade Federal de Minas Gerais Belo Horizonte Brazil; ^8^ Behavioral and Cognitive Research Group, Departamento de Clínica Médica, Faculdade de Medicina Universidade Federal de Minas Gerais Belo Horizonte Brazil; ^9^ Neurology Department Hospital de Clínicas de Porto Alegre Porto Alegre Brazil; ^10^ Unidade de Hipertensão, Instituto do Coracao (InCor), Hospital das Clinicas, Faculdade de Medicina Universidade de Sao Paulo Sao Paulo Brazil; ^11^ Unidade de Hipertensão, Disciplina de Nefrologia, Hospital das Clinicas, Faculdade de Medicina Universidade de Sao Paulo Sao Paulo Brazil

**Keywords:** cardiovascular risk factors, cognitive decline, ELSA‐Brasil, Life's Essential 8, lifestyle

## Abstract

**Background:**

Life's Simple 7, a lifestyle and cardiovascular index associated with cognition, has been updated to Life's Essential 8 (LE8) to include sleep. LE8 has been related to cardiovascular outcomes but its association with cognition is unclear.

**Method**s**:**

In this longitudinal analysis of the Brazilian Longitudinal Study of Adult Health (ELSA‐Brasil), LE8 score was based on health behaviors (diet, physical activity, nicotine exposure, and sleep health) as well as health‐related factors (body mass index, blood lipids, blood glucose, and blood pressure). Cognition was assessed in three waves, 4 years apart, using the Consortium to Establish a Registry for Alzheimer's Disease – Word List, semantic and phonemic verbal fluency, the Trail‐Making Test B (TMT‐B), and a global composite score. We used linear mixed‐model analysis, inverse probability weighting, and interaction analysis.

**Results:**

At baseline, the mean age of the study cohort was 51.4 ± 8.9 years, 56% were women, and 53% were White. Higher baseline LE8 scores were associated with slower decline in global cognition (*β* = 0.001, 95% confidence interval [CI] 0.001, 0.002; *p* < 0.001), memory (*β* = 0.001, 95% CI 0.000, 0.002; *p* = 0.013), verbal fluency (*β* = 0.001, 95% CI 0.000, 0.002; *p* = 0.003), and TMT‐B (*β* = 0.004, 95% CI 0.003, 0.005; *p* < 0.001). This association was mainly driven by LE8 health factors, particularly blood glucose and blood pressure. Age, sex, and race were modifiers of the association between LE8 and global cognitive decline (*p* < 0.001), suggesting it was more pronounced in older, male, and Black participants.

**Conclusions:**

Higher baseline LE8 scores were associated with slower global and domain‐specific cognitive decline during 8 years of follow‐up, mainly due to health factors such as blood glucose and blood pressure. Sociodemographic factors were modifiers of this association.

## INTRODUCTION

Dementia is a major public health concern, with approximately 55 million people currently living with dementia worldwide and this number expected to reach 139 million in 2050 [1]. Dementia is also one of the leading causes of mortality and disability, particularly in low‐ to middle‐income countries [2]. Engaging in a healthy lifestyle, such as eating a healthy diet, taking part in physical activity, and sleeping 7–8 h per night, has been shown to enhance cognitive health and reduce the risk of dementia [3]. Moreover, cardiovascular risk factors (CVRFs), such as hypertension, dyslipidemia, diabetes, and obesity, have been related to cognitive decline and a higher dementia risk [4, 5, 6, 7]. A clinical trial with a multidomain intervention related to lifestyle changes and CVRF control showed beneficial effects on the cognitive performance of older individuals, regardless of baseline socioeconomic and cognitive status [8].

In 2010, the American Heart Association established goals to improve cardiovascular health, also known as “Life's Simple 7” (LS7), which included seven health‐related variables: four modifiable health behaviors (smoking, body mass index [BMI], physical activity, and diet), and three health factors (total cholesterol, blood pressure, and fasting plasma glucose) [9]. Higher LS7 scores were associated with better cognitive performance in a cross‐sectional analysis of 12,271 participants aged 35–74 years from the Brazilian Longitudinal Study of Adult Health (ELSA‐Brasil), particularly in older and Black participants, and in individuals with less education [10]. A longitudinal analysis from the Atherosclerosis Risk in Communities Cohort Study (ARIC), conducted in 13,270 participants aged 45–64 years at baseline, showed that higher midlife LS7 scores were associated with better cognition in midlife, as well as slower cognitive decline over a 20‐year period, particularly in White participants [11]. Furthermore, the REasons for Geographic And Racial Differences in Stroke (REGARDS) study analyzed 30,239 participants aged 45 year and older at baseline, showing that higher LS7 scores were associated with a lower risk of cognitive impairment over a 4‐year follow‐up [12].

The American Heart Association has updated the LS7 to the “Life's Essential 8” (LE8) approach to cardiovascular health assessment. The LE8 now includes sleep health, and comprises four health behaviors (diet, physical activity, nicotine exposure, and sleep health) and four other health‐related factors (BMI, blood lipids, blood glucose, and blood pressure) and updated scores for the individual metrics [13]. The LE8 has been associated with cardiovascular outcomes mostly in White participants from the United States and United Kingdom [14, 15, 16]. Even though cardiovascular health might be slightly improving for some populational segments, cardiovascular risk markers are worsening among specific groups, particularly individuals with lower socioeconomic status, which points to the importance of conducting studies in low‐ to middle‐income countries [13]. The aim of this study was to investigate the association of baseline LE8 scores with cognitive decline in 11,390 participants from the ELSA‐Brasil study during a median follow‐up time of 8 years [17, 18, 19].

## METHODS

### Study design and participants

The ELSA‐Brasil is a longitudinal multicenter study, conducted in public institutions in six Brazilian cities: Belo Horizonte, Porto Alegre, Rio de Janeiro, Salvador, Sao Paulo, and Vitoria. The study evaluated civil servants from those centers at baseline (Wave 1; 2008–2010, *n* = 15,105), and in Wave 2 (2012–2014, *n* = 14,014), and Wave 3 (2017–2019, *n* = 12,636). In this longitudinal study, individuals were tested up to three times every 4 years (mean time between visits: 4.1 ± 0.3 years) over a median (range) follow‐up of 8 (7–9) years. The study methods and participant profiles have been described in detail elsewhere [20, 21]. At baseline, participants were aged 35–74 years, and data collection included sociodemographic variables, clinical conditions, occupational history, mental health status, cognitive assessment, and imaging and laboratory examination. Local committees at each center approved the study, which was conducted according to the Declaration of Helsinki. Before enrolling in the study, all participants gave written informed consent. ELSA‐Brasil did not include participants with cognitive impairment at baseline, and for this analysis, we excluded participants with self‐reported stroke, heart failure, coronary artery disease, and incomplete data for variables used to compute LE8, cognitive tests, or covariates.

Of the 15,105 individuals included in Wave 1, 11,390 were eligible for this analysis. In Wave 2, 104 participants died before the wave began, 647 were lost to follow‐up, and 210 had incomplete data for cognitive tests. Cognitive tests were only applied to those aged 55 years or older in Wave 2, therefore, a total of 4984 participants underwent cognitive assessment because 5445 participants were younger than 55 years. In Wave 3, all participants who attended the visit underwent cognitive assessment independently of their age. However, 178 participants died before Wave 3 began, 934 were lost to follow‐up, and 771 had incomplete data for cognitive tests. Therefore, 8756 participants completed the Wave 3 evaluation (Figure S1).

### Life's Essential 8

The LE8 score was calculated using eight metrics divided into four health behaviors (diet, physical activity, nicotine exposure, and sleep health) and four health factors (BMI, blood lipids, blood glucose, and blood pressure; Table S1) with an adaptation for the sleep metric using imputed data from Waves 2 and 3 or from a multiple imputation approach (Appendix S1). Each individual score ranged from 0 to 100 and the overall LE8 score was calculated as the unweighted average of the eight individual metrics, also ranging from 0 to 100. Higher values represented better health behavior/factor profiles. We computed score categories for the LE8 total score and for the individual metrics, categorizing the continuous variables into low (<50 points), moderate (50–79 points), and high (≥80 points) LE8 categories [13]. Additionally, we averaged the diet, physical activity, nicotine exposure, and sleep health metrics to calculate LE8 health behavior scores, and the BMI, blood lipid, blood glucose, and blood pressure metrics to calculate LE8 health factor scores. Detailed descriptions of the LE8 calculation can be found in Appendix S1.

### Cognitive function evaluation

We assessed memory using the word list immediate recall, delayed recall, and recognition tests from the Consortium to Establish a Registry for Alzheimer's Disease. Verbal fluency was assessed using semantic and phonemic tests, and executive function using the Trail‐Making Test‐B (TMT‐B) [22]. Standardized *z*‐scores were computed for each cognitive domain and for a global composite using mean and standard deviation from Wave 1. Detailed descriptions of the cognitive assessments can be found in Appendix S1.

### Other baseline variables

The sociodemographic variables included age, sex, race, and education. Race was self‐reported and classified as White, Black, Brown (mixed race of Black and White), Asian, or Indigenous. For this analysis, Black and Brown were classified as Black, and Asian and Indigenous were combined and classified as other race. Education was self‐reported and classified as lower than elementary school level, elementary school level, high school level, and college degree or higher. Depression was assessed with the Clinical Interview Schedule—Revised and classified based on the diagnostic criteria of depressive disorder from the International Classification of Diseases‐10 [23].

### Statistical analysis

For the descriptive analysis, data are presented as mean and standard deviation (SD) or median and interquartile range for continuous variables, and as relative frequencies for categorical variables. Sociodemographic and clinical variables were compared among the LE8 total score categories (low, moderate, and high).

We used linear mixed models with random intercepts for each participant and random slopes for time (using the participants' age for each wave) to investigate the relationship between baseline LE8 categories (low, moderate, and high) and cognitive decline over the study period. Each cognitive score (memory, verbal fluency, TMT‐B and global cognition) was analyzed as an independent outcome in adjusted analyses for age, sex, race, education, and depression. For the linear mixed models, positive betas indicated slower decline rates. To estimate the percentage of cognitive decline, we subtracted the slope of the moderate and high LE8 categories from the slope of the low category, divided this difference by the slope of the low category and multiplied it by 100 [24]. For sensitivity analysis, we repeated these same analyses using the next‐observation‐carried‐backward approach to impute available cognitive data from Wave 3 to missing cognitive data from Wave 2.

We investigated the effect of the interaction between sociodemographic variables and LE8 scores on the association between LE8 and cognitive decline. We conducted adjusted linear mixed model analysis that had cognitive domains as the dependent variables and added three‐way interaction terms of time and LE8 category (low, moderate, and high) with age (<60 and ≥60 years old), sex (women and men), and race (Black/Brown and White participants, excluding other races). We further stratified the linear mixed model analysis using the age, sex, and race categories.

We also investigated the associations of cognitive decline with LE8 health behaviors (by averaging the diet, physical activity, nicotine exposure, and sleep health metric scores) and LE8 health factors (by averaging the BMI, blood lipids, blood glucose, and blood pressure metric scores). Additionally, we used LE8 continuous score as the independent variable and also assessed the associations of each of the LE8 continuous individual metrics with cognitive decline in separate linear mixed models.

We used inverse probability weighting (IPW) to deal with attrition bias [25]. First, participation and survival weights were estimated as the probability of participating and being alive at each wave, based on baseline data for sociodemographics, clinical conditions, LE8 score, and cognitive performance. We then multiplied each wave weight by the previous wave weight in a cumulative manner, truncated them at the extreme percentiles (p1 and p99), and calculated the final weights by multiplying the participation and survival weights. This procedure allowed us to reweight participants who had similar profiles to those that were lost or deceased during the study period and to make attrition independent of its predictors. Analyses were conducted using the R software version 4.0.2 and the alpha level was set at 5%.

## RESULTS

Compared to included participants, excluded participants were older, more likely to be Black/Brown, less likely to be female and to have a college degree, and had lower LE8 scores and lower cognitive scores in all tests, except for the TMT‐B (Table S2). Compared to participants that were lost to follow‐up during the study period, participants that remained in the study up to Wave 3 had a better performance on all cognitive domains at baseline (Table S3).

**FIGURE 1:**
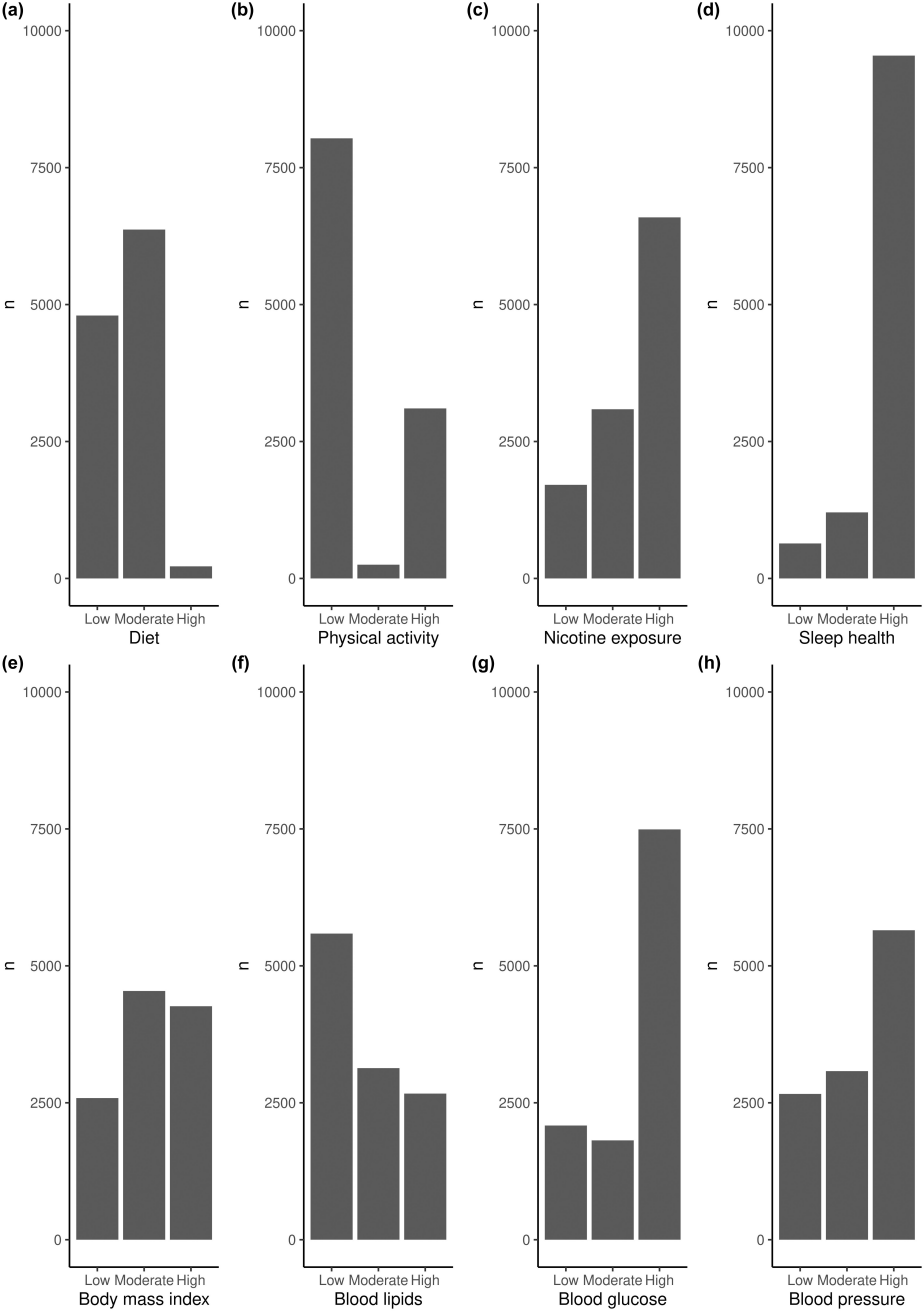
Frequency of participants by Life's Essential 8 individual metrics divided into low score (0–49), moderate score (50–79), and high score (80–100).

In 11,390 participants, the baseline mean age was 51.4 ± 8.9 years, 56% were women, 53% were White, and 55% had a college education or higher. The mean LE8 total score was 63.4 ± 13.1 (Table 1). Participants were followed for a median (interquartile range) of 8 (7–9) years. Compared to the low LE8 score group, participants with high LE8 scores were younger, more likely to be women and White, to have a college degree or higher, and to be physically active, and less likely to have depression. Participants with high LE8 scores also had a higher performance in the immediate recall, delayed recall, and recognition of the word list test, in semantic and phonemic verbal fluency, and completed the TMT‐B in a shorter time (Table 1). Regarding the distribution of the LE8 individual metrics scores, most of the participants had high scores in nicotine exposure, sleep health, blood glucose, and blood pressure, moderate scores in diet and in BMI, and low scores in physical activity and in blood lipids (Figure 1).

**TABLE 1:** Sociodemographic, clinical and cognitive characteristics of the study sample stratified by Life's Essential 8 category (*n* = 11,390).

Characteristic	Total sample	Low LE8 score	Moderate LE8 score	High LE8 score	*p* value
	*N* = 11,390	*N* = 1778	*N* = 8316	*N* = 1296	
Age, mean (SD) years^a^	51.4 (8.9)	53.8 (7.9)	51.3 (8.9)	48.4 (8.7)	<0.001
Sex: female, %^b^	55.6	48.0	56.3	61.3	<0.001
Race/ethnicity, %^b^					
Black/Brown	43.2	54.9	43.0	28.5	<0.001
White	53.5	41.7	53.7	68.0	
Other	3.4	3.3	3.3	3.6	
Education level, %^b^					
Lower than elementary school	3.7	9.2	3.1	0.7	<0.001
Elementary school	5.7	11.8	5.1	1.6	
High school	35.5	44.4	36.5	16.4	
College or higher	55.1	34.7	55.3	81.3	
Depression, %^b^	13.0	18.4	12.7	7.7	<0.001
LE8 total score, mean (SD)^a^	63.4 (13.1)	43.0 (5.5)	64.3 (8.0)	85.0 (3.9)	<0.001
LE8 diet score, mean (SD)^a^	39.9 (13.7)	35.8 (13.0)	39.8 (13.6)	46.6 (13.1)	<0.001
LE8 physical activity score, mean (SD)^a^	28.3 (43.6)	2.6 (14.7)	24.5 (41.4)	87.9 (29.3)	<0.001
LE8 nicotine exposure score, mean (SD)^a^	74.1 (33.2)	50.2 (39.8)	76.3 (31.0)	92.7 (13.8)	<0.001
LE8 sleep health score, mean (SD)^a^	90.9 (17.0)	83.1 (23.9)	91.8 (15.5)	95.8 (9.2)	<0.001
LE8 BMI score, mean (SD)^a^	71.0 (28.6)	44.9 (28.6)	73.2 (26.3)	92.2 (14.5)	<0.001
LE8 blood lipid score, mean (SD)^a^	53.0 (29.7)	34.5 (25.3)	53.7 (28.7)	74.3 (26.3)	<0.001
LE8 blood glucose score, mean (SD)^a^	81.8 (26.7)	55.7 (29.1)	85.0 (24.1)	97.0 (11.0)	<0.001
LE8 blood pressure score, mean (SD)^a^	68.0 (33.5)	37.3 (31.4)	70.5 (31.4)	93.4 (14.8)	<0.001
Immediate word list recall score (number of words), mean (SD) ^a^	21.3 (3.8)	20.3 (3.9)	21.3 (3.8)	22.6 (3.4)	<0.001
Delayed word list recall score (number of words), mean (SD)^a^	7.0 (1.9)	6.5 (2.0)	7.1 (1.9)	7.7 (1.7)	<0.001
Word list recognition score (number of words), mean (SD)^a^	9.6 (0.9)	9.4 (1.1)	9.6 (0.8)	9.7 (0.6)	<0.001
Semantic verbal fluency score (number of words), mean (SD)^a^	18.8 (5.2)	17.6 (5.1)	18.8 (5.2)	20.2 (5.0)	<0.001
Phonemic verbal fluency score (number of words), mean (SD)^a^	12.7 (4.4)	11.7 (4.4)	12.8 (4.3)	14.0 (4.1)	<0.001
Trail‐Making Test B, (seconds), mean (SD)^a^	122.5 (84.8)	152.0 (103.9)	120.6 (81.9)	94.6 (57.2)	<0.001

*Note*: Low LE8 score: 0–49; Moderate LE8 score: 50–79; High LE8 score: 80–100.

Abbreviations: BMI, body mass index; LE8, Life's Essential 8; SD, standard deviation.

^a^ One‐way analysis of variance.

^b^ Chi‐squared test.

In fully adjusted models using LE8 as a continuous score, we observed that higher baseline LE8 total scores were associated with slower decline in memory (*β* = 0.001, 95% confidence interval [CI] 0.000, 0.002; *p* = 0.013), verbal fluency (*β* = 0.001, 95% CI 0.000, 0.002; *p* = 0.003), executive function (*β* = 0.004, 95% CI 0.003, 0.005; *p* < 0.001) and global cognition (*β* = 0.001, 95% CI 0.001, 0.002; *p* < 0.001 [Table 2]). Sensitivity analysis using next observation carried backward to impute missing cognitive data from Wave 2 showed similar results (Table S4).

**TABLE 2:** Association between baseline Life's Essential 8 score (continuous and categorical variables) and cognitive change over the study period (*n* = 11,390).

	Unadjusted		Model 1		
	*β* (95% CI)		*β* (95% CI)		Difference^a^, %
Memory					
LE8 continuous		*p* value		*p* value	
LE8 score*time	0.001 (0.000; 0.002)	0.005	0.001 (0.000; 0.002)	0.013	–
LE8 category		*p* for trend		*p* for trend	
Low score*time	Reference		Reference		Reference
Moderate score*time	0.001 (−0.002; 0.005)	0.069	0.000 (−0.002; 0.004)	0.088	6%
High score*time	0.005 (0.000; 0.010)		0.004 (0.000; 0.009)		33%
Verbal fluency					
LE8 continuous		*p* value		*p* value	
LE8 score*time	0.002 (0.001; 0.003)	<0.001	0.001 (0.000; 0.002)	0.003	–
LE8 category		*p* for trend		*p* for trend	
Low score*time	Reference		Reference		Reference
Moderate score*time	0.007 (0.003; 0.010)	<0.001	0.004 (0.001; 0.007)	0.007	28%
High score*time	0.010 (0.005; 0.014)		0.006 (0.002; 0.011)		42%
Executive function					
LE8 continuous		*p* value		*p* value	
LE8 score*time	0.005 (0.004; 0.006)	<0.001	0.004 (0.003; 0.005)	<0.001	–
LE8 category		*p* for trend		*p* for trend	
Low score*time	Reference		Reference		Reference
Moderate score*time	0.012 (0.008; 0.016)	<0.001	0.009 (0.005; 0.013)	<0.001	34%
High score*time	0.021 (0.015; 0.027)		0.016 (0.011; 0.021)		61%
Global cognition					
LE8 continuous		*p* value		*p* value	
LE8 score*time	0.002 (0.001; 0.002)	<0.001	0.001 (0.001; 0.002)	<0.001	–
LE8 category		*p* for trend		*p* for trend	
Low score*time	Reference		Reference		Reference
Moderate score*time	0.003 (0.001; 0.006)	<0.001	0.002 (0.000; 0.004)	0.002	14%
High score*time	0.007 (0.004; 0.011)		0.005 (0.002; 0.009)		42%

*Note*: LE8 score: Life's Essential 8 score divided by 10: low score: 0–49; moderate score: 50–79; high score: 80–100. Model 1: linear mixed models with random intercepts and slopes adjusted for age at baseline, sex, race/ethnicity, education, and depression. Inverse probability weighting for censoring was used to account for attrition bias.

Abbreviation: CI, confidence interval.

^a^ Difference between the slope of each category of the LE8 score and the slope of the first category divided by the slope of the first category multiplied by 100.

Compared to participants with low LE8 scores, participants with moderate LE8 scores had a 14% slower global cognitive decline (*β* = 0.002, 95% CI 0.000, 0.004; *p* for trend = 0.002), while participants with high LE8 scores had a 42% slower global cognitive decline (*β* = 0.005, 95% CI 0.002, 0.009; *p* for trend = 0.002). Moreover, compared to participants with low LE8 scores, participants with moderate LE8 scores had a 28% slower verbal fluency decline (*β* = 0.004, 95% CI 0.001, 0.007; *p* for trend = 0.007) while participants with high LE8 scores had a 42% slower verbal fluency decline (*β* = 0.006, 95% CI 0.002, 0.011; *p* for trend = 0.007). Compared to participants with low LE8 score, participants with moderate LE8 scores had a 34% slower executive function decline (*β* = 0.009, 95% CI 0.005, 0.013; *p* for trend <0.001), while participants with high LE8 scores had a 61% slower executive function decline (*β* = 0.016, 95% CI 0.011, 0.021; *p* for trend <0.001 [Table 2 and Figure S2]). We observed similar results in sensitivity analysis using imputed cognitive data in Wave 2 (Table S4).

**FIGURE 2:**
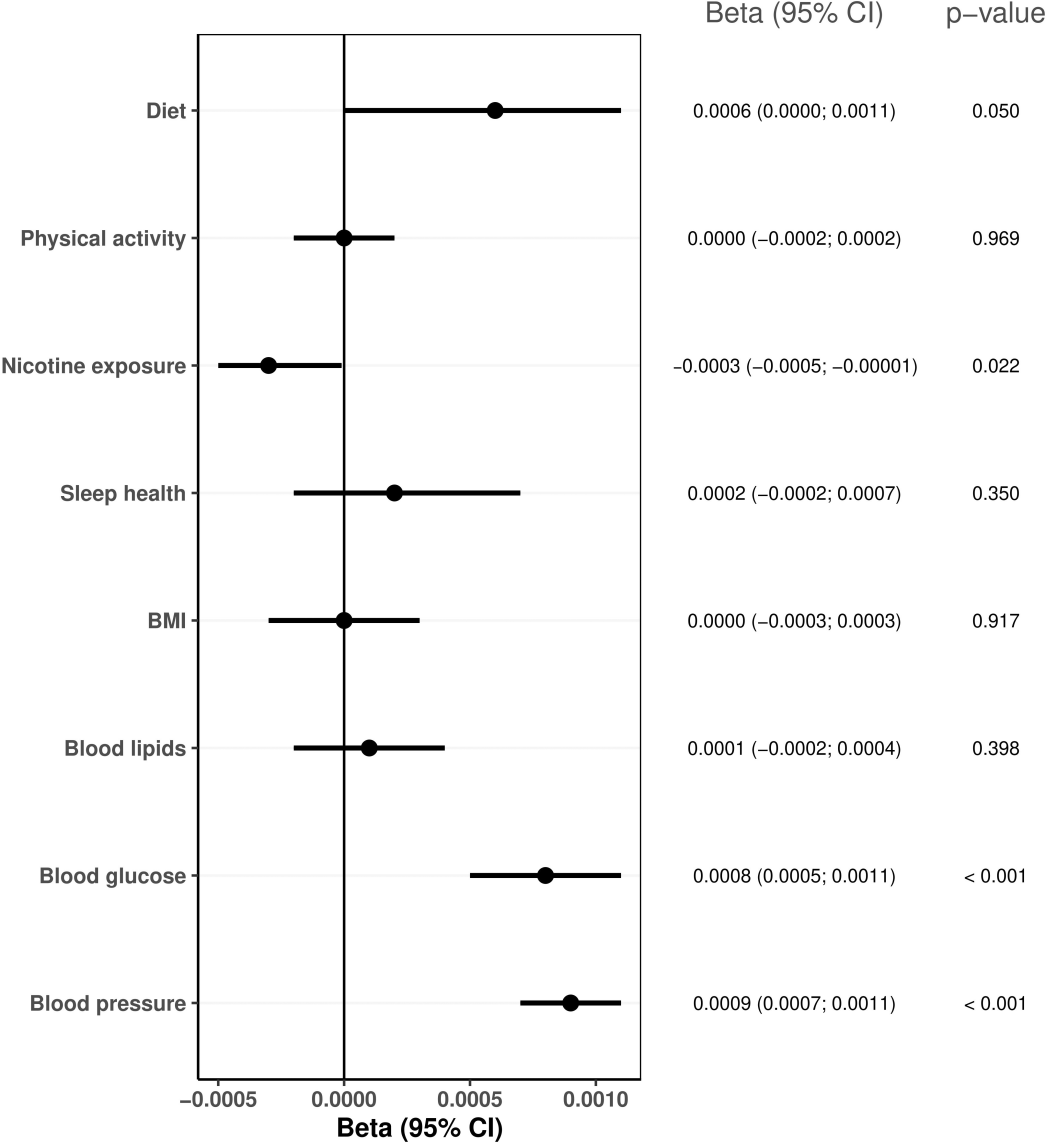
Association between Life's Essential 8 individual metrics (continuous scores) and cognitive decline over the study period. Linear mixed models with random intercepts and slopes adjusted for age at baseline, sex, education, race, and depression. Positive coefficients indicate slower decline rates.

Higher scores in the combined LE8 health factors were associated with slower cognitive decline in global cognition and in the specific cognitive domains, while higher scores in the combined LE8 health behaviors were only associated with slower decline in executive function (*p* < 0.019; Table 3). Regarding the individual continuous metrics, higher blood glucose scores (i.e., lower blood glucose levels; *β* = 0.0008, 95% CI 0.0005, 0.0011; *p* < 0.001) and higher blood pressure scores (i.e., lower blood pressure levels; *β* = 0.0009, 95% CI 0.0007, 0.0011; *p* < 0.001) were associated with slower global cognitive decline, while higher nicotine exposure scores (i.e., lower nicotine exposure; *β* = −0.0003, 95% CI −0.0005, −0.00001; *p* = 0.022) were associated with faster global cognitive decline (Figure 2). Tables S5–S12 present LE8 individual metrics categories in relation to cognitive decline.

**TABLE 3:** Association of baseline health behavior and health factor metrics from Life's Essential 8 with cognitive change over the study period (*n* = 11,390).

	Health behavior metrics		Health factor metrics	
	*β* (95% CI)	*p* for trend	*β* (95% CI)	*p* for trend
Memory				
Low*time	Reference		Reference	
Moderate*time	0.001 (−0.002; 0.003)	0.544	0.004 (0.001; 0.008)	0.001
High*time	−0.001 (−0.004; 0.002)		0.006 (0.003; 0.010)	
Verbal fluency				
Low*time	Reference		Reference	
Moderate*time	0.000 (−0.002; 0.002)	0.990	0.005 (0.002; 0.008)	<0.001
High*time	0.000 (−0.002; 0.003)		0.008 (0.004; 0.011)	
Executive function				
Low*time	Reference		Reference	
Moderate*time	0.002 (−0.001; 0.005)	0.019	0.009 (0.005; 0.013)	<0.001
High*time	0.006 (0.002; 0.010)		0.014 (0.010; 0.018)	
Global cognition				
Low*time	Reference		Reference	
Moderate*time	0.000 (−0.002; 0.002)	0.969	0.005 (0.003; 0.007)	<0.001
High*time	0.000 (−0.002; 0.002)		0.007 (0.005; 0.010)	

*Note*: Health behavior metrics: average of diet, physical activity, nicotine exposure, and sleep health metrics scores divided by 10. Health factor metrics: average of body mass index, blood lipids, blood glucose, and blood pressure metrics scores divided by 10. Low score: 0–49; moderate score: 50–79; high score: 80–100. Linear mixed models with random intercepts and slopes adjusted for age at baseline, sex, race/ethnicity, education, and depression. Inverse probability weighting for censoring was used to account for attrition bias.

**FIGURE 3:**
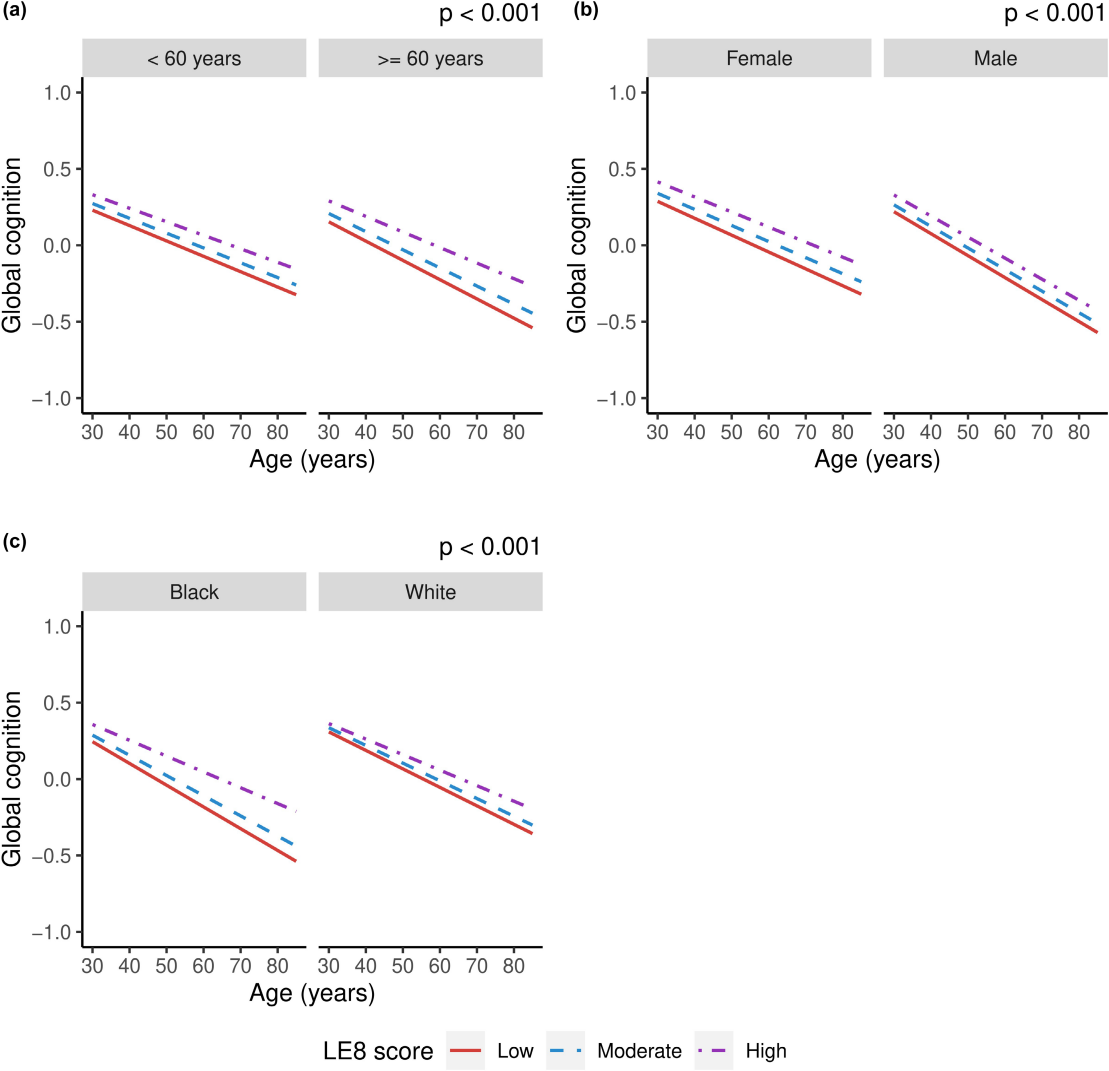
Association between Life's Essential 8 (LE8) total score categories (divided into low score [0–49], moderate score [50–79], and high score [80–100]) and global cognitive decline over the study period stratified by: (a) age: <60 and ≥60 years old; (b) female and male participants; and (c) Black and White participants. Linear mixed models with random intercepts and slopes adjusted for age at baseline, sex, education, race, and depression (*p* for the interaction terms).

Interaction analysis showed that the association between LE8 category and global cognitive decline was modified by age, sex, and race (*p* < 0.001 for all interactions), suggesting the association is more pronounced in older, male, and Black participants (Figure 3). Stratified analysis showed that LE8 category was associated with global cognitive decline in men (*p* = 0.015), but not in women (*p* = 0.154). We did not find associations between LE8 categories and global cognitive decline in stratified analyses by age and race (Table S13).

## DISCUSSION

In 11,390 participants from ELSA‐Brasil, we observed that higher scores in the LE8 were associated with slower decline in global cognition and in specific cognitive domains (i.e., memory, verbal fluency, and executive functioning) over 8 years of follow‐up. We also observed that the combined health factors were associated with slower decline in global cognition and in all evaluated cognitive domains, while the combined health behaviors were only associated with slower decline in executive function. Age, sex, and race were modifiers on the association between LE8 and global cognitive decline, suggesting that older, male, and Black participants had the most benefit of higher LE8 scores over the study period.

Higher LE8 scores were associated with lower dementia risk, better cognitive performance, and larger total brain and hippocampal volumes in 316,669 participants from the UK Biobank study [26]. Furthermore, LE8 scores were shown to be associated with CVRFs and cardiovascular disease (CVD) in previous studies [14, 16, 27], which, in turn, were associated with cognitive impairment and dementia [28, 29]. A study including 250,825 participants from the UK Biobank used a modified version of the LE8 to predict the risk of developing ischemic heart disease, myocardial infarction, stroke, and heart failure. Compared to individuals in the highest LE8 quartile, participants in the lowest quartile had a more than twofold higher risk of developing one of the CVD outcomes [16]. A cross‐sectional study conducted in obese individuals evaluated the participants’ anthropometric and biochemical profiles and verified that low LE8 scores were associated with higher blood pressure (peripheral and central), arterial stiffness, and endothelial dysfunctions [27].

The addition of sleep measurements seems to improve the predictive ability of the LS7 index. The Multi‐Ethnic Study of Atherosclerosis (MESA) Sleep Study observed that, even though the LS7 was related to CVD prevalence, it was only possible to predict CVD incidence with the addition of a sleep metric to LS7 [30]. Additionally, LE8 scores seemed to be more sensitive than LS7 to classify cardiovascular health in young adults [14]. In a previous cross‐sectional analysis of ELSA‐Brasil, higher LS7 scores were associated with better performance in memory, verbal fluency, and global cognition, but not in executive function [10]. However, in the present longitudinal analysis using LE8 scores as predictors, we observed that higher LE8 score was associated with slower decline in global cognition and all cognitive domains.

In our study, CVRFs seemed to play a stronger role than lifestyle factors in the association between LE8 and cognitive decline. This finding highlights the importance of understanding the pathway of modifiable risk factors for dementia, on which lifestyle factors might be downstream predictors of cognitive decline, mediated by more upstream predictors such as CVRFs [31]. Lifestyle behaviors, such as unhealthy eating, physical inactivity, tobacco use, and poor‐quality sleep, are well known to be related to CVD [30, 32]. Furthermore, CVRFs, such as obesity, high cholesterol, high blood glucose, and high blood pressure, are important mediators between lifestyle factors and CVD, as they lead to increased arterial wall inflammation and stiffness, increased carotid intima‐media thickness, and atherosclerotic plaques deposition [32, 33, 34, 35]. which have also been associated with cognitive decline and dementia risk [36, 37, 38, 39]. These arterial changes lead to dysregulated cerebral blood flow, cerebral hypoperfusion, brain infarctions, white matter hyperintensities, and capillary wall degeneration [40, 41].

Individual LE8 metrics have been associated with cognitive performance in previous studies. The Northern Manhattan study evaluated 1290 stroke‐free participants from a multiethnic community, and found that metabolic syndrome, particularly high blood pressure, was associated with poor cognitive performance [42]. We observed that lower blood pressure and blood glucose levels were associated with slower cognitive decline. Regarding lifestyle behaviors, the Hellenic Longitudinal Investigation of Aging and Diet (HELIAD) study investigated the association between a total lifestyle index (assessing adherence to Mediterranean diet, sleep duration, physical activity, and engagement in daily life activities) and cognitive decline or dementia risk in 1018 community‐dwelling older adults over 3 years of follow‐up. Participants who did not develop dementia had higher scores on the total lifestyle index and for all the individual lifestyle factors, except for sleep duration [43]. In our study, higher scores on the diet metric were marginally associated with slower cognitive decline, and less nicotine exposure was associated with faster cognitive decline. The finding of nicotine exposure being associated with better cognitive outcomes has been previously attributed to selection bias. Even though we addressed this issue in our statistical approach using IPW, some residual bias may still be present [44]. On the other hand, even though tobacco use has well‐known detrimental effects on cardiovascular health, previous studies showed conflicting results on the association between nicotine exposure and cognition [45].

Our findings on LE8 being a predictor of cognitive decline were consistent. ELSA‐Brasil is a large cohort study with a median follow‐up of 8 years, which gave us the opportunity to investigate the association between baseline LE8 scores and cognitive performance over time. Furthermore, this study was conducted in a low‐ to middle‐income country and included a racially admixed sample, allowing us to analyze the implications of the new scoring on cognitive decline in a diverse setting [13, p.8]. Another strength of our study was the inclusion of middle‐aged individuals, making it possible to investigate the relationship between lifestyle or cardiovascular risk in midlife and cognitive decline long before dementia is fully established.

The study also has some limitations. Although a wide range of variables were used to adjust the analyses, residual confounding may still be present. In addition, due to the study design, we did not have information on hours of sleep for Wave 1, and had to impute this using hours of sleep from Waves 2 and 3 and other related variables from Wave 1. Furthermore, participants that were lost to follow‐up had worse cognitive performance at baseline than participants who remained in the study up to Wave 3. However, we partially addressed this bias using IPW. Another limitation is the absence of neuroimaging and apolipoprotein E genotyping, as we were not able to investigate potential brain lesions related to our findings or conduct stratified analysis by apolipoprotein E allele ε4.

In conclusion, in this large longitudinal study with an admixed population from a low‐ to middle‐income country, higher baseline LE8 scores were associated with slower global and domain‐specific decline after 8 years of follow‐up. This association was mainly attributable to CVRFs, rather than to lifestyle factors. Our findings reinforce the importance of controlling for CVRFs in midlife to prevent cognitive decline and decrease dementia risk.

## AUTHOR CONTRIBUTIONS

**Naomi Vidal Ferreira:** Conceptualization; writing – original draft; methodology; writing – review and editing; formal analysis; visualization. **Natalia Gomes Gonçalves:** Methodology; formal analysis; writing – review and editing; visualization. **Claudia Szlejf:** Writing – review and editing; validation. **Maria Inês Schmidt:** Validation; writing – review and editing. **Sandhi Maria Barreto:** Validation; writing – review and editing. **Paulo Caramelli:** Validation; writing – review and editing. **Natan Feter:** Validation; writing – review and editing. **Raphael Machado Castilhos:** Validation; writing – review and editing; methodology; formal analysis. **Paulo Lotufo:** Funding acquisition; writing – review and editing; validation; investigation; project administration; data curation. **Claudia Kimie Suemoto:** Supervision; conceptualization; validation; methodology; writing – review and editing.

## FUNDING INFORMATION

The ELSA‐Brasil was supported by the Brazilian Ministry of Health (Science and Technology Department) and the Brazilian Ministry of Science and Technology (FINEP, Financiadora de Estudos e Projetos and CNPq, National Research Council). Grants: 01 060010.00 RS, 01 060212.00 BA, 01 060300.00 ES, 01 060278.00 MG, 01 060115.00 SP, 01 060071.00 RJ.

## CONFLICT OF INTEREST STATEMENT

None.

Abbreviation: CI, confidence interval.

## Supporting information


Appendix S1.

## Data Availability

The data that support the findings of this study are available from the corresponding author upon reasonable request.
