# Supplementary material for: Optimal cardiovascular health is associated with slower cognitive decline
Source: Eur J Neurol. 2023 Nov 28;31(2):e16139. doi: 10.1111/ene.16139 (PMC11235920; doi:10.1111/ene.16139)
Supplement: Supplementary file 1 — Appendix S1. [file ENE-31-e16139-s001.zip › Supplemental material_2.docx]

**Supplemental Material**

**Appendix 1**

**Supplemental Methods**

*Calculation of the Life's Essential 8 score*

We measured the participants' dietary patterns using a 114-item Food Frequency Questionnaire that assessed the usual consumption of each food item during the last 12 months.[^1^](https://www.zotero.org/google-docs/?jn1as5) The diet metric was calculated based on the Mediterranean Eating Pattern for Americans (MEPA) 16-item tool, which assessed how frequently the participants consumed 16 food items (olive oil, green leafy vegetables, other vegetables, berries, other fruit, meat, fish, chicken, cheese, butter/cream, beans, whole grains, sweets and pastries, nuts, fast food, and alcohol). Using the Food Frequency Questionnaire consumption report, 1 point was attributed to the ideal intake of each food/food group, and the total MEPA score ranged from 0 to 16. The final diet metric classified the MEPA score into a 0 to 100 range.

We measured the frequency, time, and intensity of physical activity using the International Physical Activity Questionnaire (IPAQ),[^2^](https://www.zotero.org/google-docs/?4Cnb7I) and the physical activity metric was calculated using minutes of moderate or greater intensity activity, which were classified into a 0 to 100 range.

The nicotine exposure metric was classified using the information on combustible tobacco use (current or past), time since quitting smoking, and second-hand smoking exposure. The nicotine exposure score ranged from 0 to 100.

Due to the study design, the information on hours of sleep was collected only in waves 2 and 3. For participants who had the same number of hours of sleep per night for waves 2 and 3, we imputed that number for wave 1. For everybody else, we imputed the hours of sleep per night for wave 1 using Multiple Imputation by Chained Equations (MICE) based on age, sex, alcohol use, smoking status, physical activity, body mass index (BMI), and depression to estimate hours of sleep.[^3^](https://www.zotero.org/google-docs/?eK5QGy) The sleep health metric was based on the participants' average hours of sleep per night and ranged from 0 to 100.

BMI was calculated as the measured weight (kg) divided by squared measured height (m^2^) and the metric classified BMI values into a 0 to 100 range.

Non-HDL cholesterol was calculated by subtracting HDL-cholesterol from total cholesterol. The blood lipid metric used information on non-HDL cholesterol and the use of lipid-lowering drugs to calculate a score that ranged from 0 to 100.

Information on previous diabetes diagnoses and measured glycated hemoglobin levels were used to compute the blood glucose metric score that ranged from 0 to 100.

We collected information on antihypertensive treatment and measured systolic and diastolic blood pressures. The blood pressure metric used those three variables to calculate a 0 to 100 score.

*Cognitive function evaluation*

Cognitive performance was assessed during an interview conducted by trained researchers in a quiet room with adequate lighting and temperature.[^4^](https://www.zotero.org/google-docs/?4vXYfO) The Consortium to Establish a Registry for Alzheimer’s Disease (CERAD) Word List assesses verbal learning and memory (immediate, delayed, and recognition). For the immediate verbal memory task, the participants were required to read a list with 10 words printed on cards. The 10-word list is presented three times, and the participants were required to read and recall the words immediately after each presentation. For the delayed memory task, the participants were required to recall the 10 words after a 5-minute interval, without a new card presentation. For the recognition task, the participant was asked to recognize the 10 previously presented words among 10 other distracting words (words not presented before). The maximum score for each task was the number of words recalled (higher scores are better).

The verbal fluency test assesses executive function and language. For semantic verbal fluency, the participants were required to say as many names as they could in one minute within a certain semantic category (the animal category was used in waves 1 and 3, while the flora category was used in wave 2, for which the participant is required to say the names of vegetables, flowers, or trees). For phonemic verbal fluency, the participants were required to say as many words beginning with a particular letter as they could (letter F was used in waves 1 and 3 and letter A was used in wave 2). The variation in verbal fluency categories between waves 1 and 2 was used to attenuate learning effects, and test scores from wave 2 were harmonized to make them comparable to scores from waves 1 and 3.[^5,6^](https://www.zotero.org/google-docs/?MVU8vN) The score for each verbal fluency test (semantic and phonemic) was the total number of words the participant was able to generate.

The Trail Making Test B assesses executive function (cognitive flexibility and psychomotor speed) and visual-spatial organization. Participants were required to draw a line to connect numbers and letters (which are placed on a paper sheet in a non-organized manner) alternating between ascending numbers (from 1 to 13) and letters in alphabetical order (A to L) so that the line connects the numbers and letters in the following order: 1, A, 2, B, 3, C, etc. They were required to draw this line as quickly as possible. Trail Making Test B score is computed as the time in seconds the participant took to complete the test (longer times indicate worse cognitive performance). Trail Making Test B scores were multiplied by -1 so that higher scores mean better performance like the other tests.

Cognitive performance z-scores were created for each test by subtracting the sample mean at wave 1 from the participant's score on each wave and dividing it by wave 1 standard deviation for that test. Therefore, a z-score of -1 at waves 2 or 3 represented a decline of one-standard deviation compared to the sample mean performance at wave 1.[^6^](https://www.zotero.org/google-docs/?8yiZrj) We then created memory and verbal fluency scores for each wave by averaging the three memory and two verbal fluency scores, respectively. Furthermore, a global cognitive score for each wave was calculated by averaging the scores from the six cognitive tests.

**Supplemental Figures and Tables**


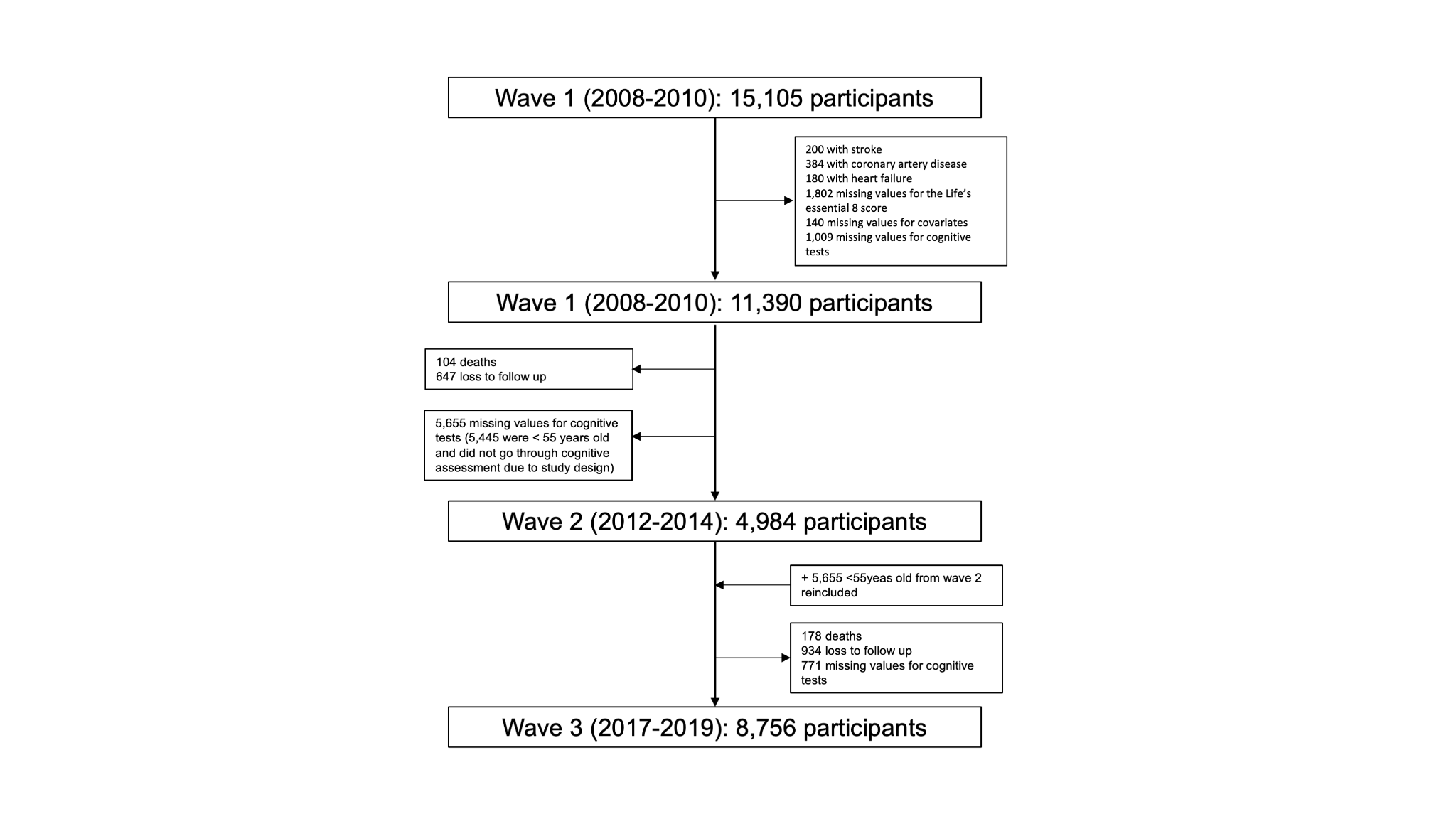


**Supplemental Figure 1.** Inclusion diagram.

**
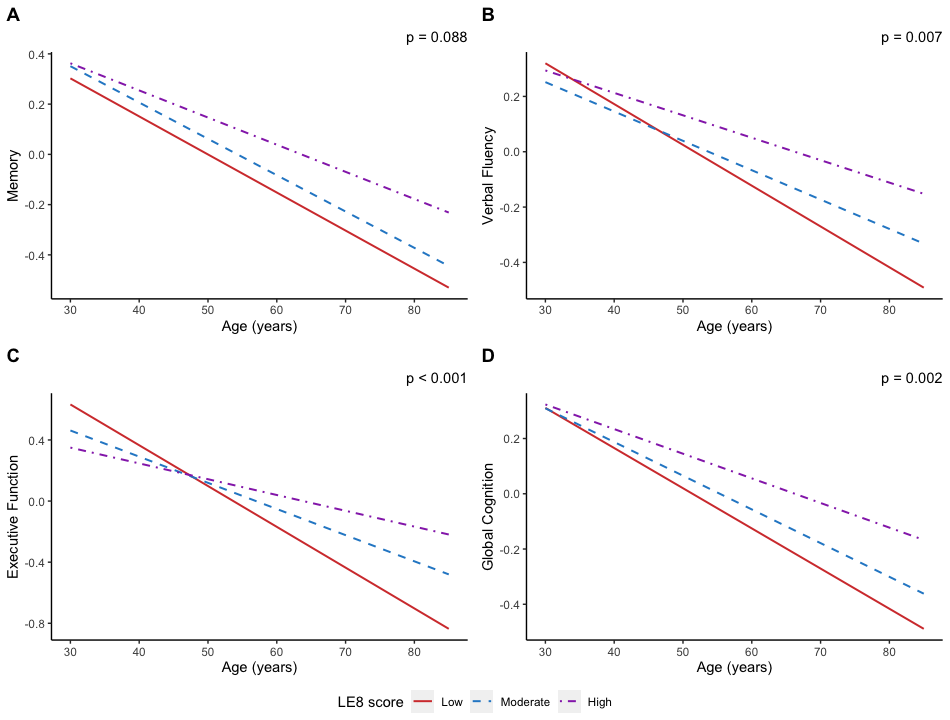
**

**Supplemental Figure 2.** Life's essential 8 total score categories (divided into low score (0-49), moderate score (50-79), and high score (80-100)), and its association with cognitive decline over the study period for the whole sample. Linear mixed models with random intercepts and slopes adjusted for age at baseline, sex, education, race, and depression (p for trend for the interaction terms).

**Supplemental Table 1.** Life’s essential 8 individual metrics calculation.

| **Health behaviors** | Diet | **Points:** | **Metric: MEPA score^a^.**  **Level:** |
| --- | --- | --- | --- |
|  |  | 100 | 15-16 |
|  |  | 80 | 12-14 |
|  |  | 50 | 8-11 |
|  |  | 25 | 4-7 |
|  |  | 0 | 0-3 |
|  | Physical activity | **Points:** | **Metric: Minutes of moderate- (or greater) intensity activity per week.**  **Minutes:** |
|  |  | 100 | ≥150 |
|  |  | 90 | 120–149 |
|  |  | 80 | 90–119 |
|  |  | 60 | 60–89 |
|  |  | 40 | 30–59 |
|  |  | 20 | 1–29 |
|  |  | 0 | 0 |
|  | Nicotine exposure | **Points:** | **Metric: Combustible tobacco use or inhaled NDS use; or secondhand smoke exposure.**  **Status:** |
|  |  | 100 | Never smoker |
|  |  | 75 | Former smoker, quit ≥5 y |
|  |  | 50 | Former smoker, quit 1–<5 y |
|  |  | 25 | Former smoker, quit <1 y, or currently using inhaled NDS |
|  |  | 0 | Current smoker |
|  |  | Subtract 20 points (unless score is 0) for living with active indoor smoker in home |  |
|  | Sleep health | **Points:** | **Metric: Average hours of sleep per night.**  **Level:** |
|  |  | 100 | 7 to <9 |
|  |  | 90 | 9 to <10 |
|  |  | 70 | 6 to <7 |
|  |  | 40 | 5 to <6 or ≥ 10 |
|  |  | 20 | 4 to <5 |
|  |  | 0 | <4 |
| **Health**  **factors** | BMI | **Points:** | **Metric: BMI (kg/m2).**  **Level:** |
|  |  | 100 | <25 |
|  |  | 70 | 25.0–29.9 |
|  |  | 30 | 30.0–34.9 |
|  |  | 15 | 35.0–39.9 |
|  |  | 0 | ≥40.0 |
|  | Blood lipids | **Points:** | **Metric: Non–HDL cholesterol (mg/dL).**  **Level:** |
|  |  | 100 | <130 |
|  |  | 60 | 130–159 |
|  |  | 40 | 160–189 |
|  |  | 20 | 190–219 |
|  |  | 0 | ≥220 |
|  |  | If drug-treated level, subtract 20 points |  |
|  | Blood glucose | **Points:** | **Metric: HbA1c (%).**  **Level:** |
|  |  | 100 | No history of diabetes and HbA1c <5.7 |
|  |  | 60 | No diabetes and HbA1c 5.7–6.4 (prediabetes) |
|  |  | 40 | Diabetes with HbA1c <7.0 |
|  |  | 30 | Diabetes with HbA1c 7.0–7.9 |
|  |  | 20 | Diabetes with HbA1c 8.0–8.9 |
|  |  | 10 | Diabetes with Hb A1c 9.0–9.9 |
|  |  | 0 | Diabetes with HbA1c ≥10.0 |
|  | Blood pressure | **Points:** | **Metric: Systolic and diastolic BPs (mmHg).**  **Level:** |
|  |  | 100 | <120 and <80 (optimal) |
|  |  | 75 | 120–129 and <80 (elevated) |
|  |  | 50 | 130–139 or 80–89 (stage 1 hypertension) |
|  |  | 25 | 140–159 or 90–99 |
|  |  | 0 | ≥160 or ≥100 |
|  |  | Subtract 20 points if treated level |  |

Source – adapted from LLOYD-JONES, Donald M. et al. Life’s essential 8: updating and enhancing the American Heart Association’s construct of cardiovascular health: a presidential advisory from the American Heart Association. Circulation, v. 146, n. 5, p. e18-e43, 2022.

^a^Mediterranean Eating Pattern for Americans score: based on the frequency and/or amount the participants consumed olive oil, green leafy vegetables, other vegetables, berries, other fruit, meat, fish, chicken, cheese, butter/cream, beans, whole grains, sweets and pastries, nuts, fast food, and alcohol. Range: 0 to 16.

NDS indicates nicotine-delivery system; BMI: body mass index; Non-HDL: non-high-density lipoprotein cholesterol, calculated as total cholesterol minus high density lipoprotein cholesterol; HbA1c: hemoglobin A1c; BPs: blood pressures.

**Supplemental Table 2.** Comparison between participants that were included and excluded from this analysis.

| **Characteristics** | **Included participants (n=11,390)** | **Excluded participants (n=3,715)** | **P-value** |
| --- | --- | --- | --- |
| **Age (years old), mean (SD)*** | 51.4 (8.9) | 54.3 (9.2) | <0.001 |
| **Sex (female), %**† | 55.6 | 50.7 | <0.001 |
| **Race/ethnicity, %**† |  |  | <0.001 |
| Black/Brown | 43.2 | 48.1 |  |
| White | 53.5 | 47.7 |  |
| Other | 3.4 | 4.2 |  |
| **Education, %**† |  |  | <0.001 |
| Less than elementary school | 3.7 | 12.6 |  |
| Elementary school | 5.7 | 10.1 |  |
| High school | 35.5 | 32.1 |  |
| College or more | 55.1 | 45.7 |  |
| **Depression, %**† | 13.0 | 13.7 | 0.267 |
| **LE8 total score, mean (SD)*** | 63.4 (13.1) | 57.9 (12.7) | <0.001 |
| LE8 diet, mean (SD)* | 39.9 (13.7) | 40.1 (13.7) | 0.400 |
| LE8 physical activity, mean (SD)* | 28.3 (43.6) | 20.6 (38.9) | <0.001 |
| LE8 nicotine exposure, mean (SD)* | 74.1 (33.2) | 72.3 (33.5) | 0.006 |
| LE8 sleep health, mean (SD)* | 90.9 (17.0) | 90.2 (18.2) | 0.049 |
| LE8 BMI, mean (SD)* | 71.0 (28.6) | 70.0 (28.4) | 0.063 |
| LE8 blood lipid, mean (SD)* | 53.0 (29.7) | 52.9 (29.9) | 0.311 |
| LE8 blood glucose, mean (SD)* | 81.8 (26.7) | 77.5 (29.1) | <0.001 |
| LE8 blood pressure, mean (SD)* | 68.0 (33.5) | 61.6 (34.7) | <0.001 |
| **Immediate word list recall (number of words), mean (SD)*** | 21.3 (3.8) | 20.6 (4.2) | <0.001 |
| **Delayed word list recall (number of words), mean (SD)*** | 7.0 (1.9) | 6.7 (2.1) | <0.001 |
| **Word list recognition (number of words), mean (SD)*** | 9.6 (0.9) | 9.5 (1.1) | <0.001 |
| **Semantic verbal fluency (number of words), mean (SD)*** | 18.8 (5.2) | 17.4 (5.5) | <0.001 |
| **Phonemic verbal fluency (number of words), mean (SD)*** | 12.7 (4.4) | 11.6 (4.9) | <0.001 |
| **Trail Making Test B (seconds), mean (SD)*** | 122.5 (84.8) | 123.8 (92.2) | 0.912 |

LE8: Life's essential 8; SD: standard deviation; BMI: body mass index.

*Unpaired T Test; †Chi-square Test.

**Supplemental Table 3.** Association between baseline Life’s Essential 8 score (continuous and categories) and cognitive change over the study period using next observation carried backward to impute missing cognitive data from wave 2.

|  | **Unadjusted** | | **Model 1** | |  |
| --- | --- | --- | --- | --- | --- |
|  | **β (95% CI)** |  | **β (95% CI)** |  | **Difference**^a^**, %** |
| **MEMORY** | | | | |  |
| **LE8 continuous** |  | **P-value** |  | **P-value** |  |
| LE8 score*Time | 0.0013 (0.0004; 0.0022) | 0.004 | 0.0010 (0.0001; 0.0018) | 0.013 | - |
| **LE8 categories** |  | **P for trend** |  | **P for trend** |  |
| Low score*Time | Reference |  | Reference |  | Reference |
| Moderate score*Time | 0.0017 (-0.0017; 0.0050) | 0.062 | 0.0008 (-0.0024; 0.0041) | 0.166 | 6% |
| High score*Time | 0.0053 (0.0007; 0.0098) |  | 0.0039 (-0.0005; 0.0082) |  | 28% |
| **VERBAL FLUENCY** | | | | |  |
| **LE8 continuous** |  | **P-value** |  | **P-value** |  |
| LE8 score*Time | 0.0020 (0.0012; 0.0029) | <0.001 | 0.0013 (0.0005; 0.0021) | 0.003 | - |
| **LE8 categories** |  | **P for trend** |  | **P for trend** |  |
| Low score*Time | Reference |  | Reference |  | Reference |
| Moderate score*Time | 0.0069 (0.0037; 0.0102) | <0.001 | 0.0044 (0.0013; 0.0074) | 0.003 | 30% |
| High score*Time | 0.0103 (0.0059; 0.0146) |  | 0.0068 (0.0027; 0.0109) |  | 47% |
| **EXECUTIVE FUNCTION** | | | | |  |
| **LE8 continuous** |  | **P-value** |  | **P-value** |  |
| LE8 score*Time | 0.0043 (0.0033; 0.0053) | <0.001 | 0.0034 (0.0024; 0.0043) | <0.001 | - |
| **LE8 categories** |  | **P for trend** |  | **P for trend** |  |
| Low score*Time | Reference |  | Reference |  | Reference |
| Moderate score*Time | 0.0114 (0.0075; 0.0153) | <0.001 | 0.0091 (0.0056; 0.0127) | <0.001 | 36% |
| High score*Time | 0.0201 (0.0149; 0.0253) |  | 0.0157 (0.0110; 0.0205) |  | 62% |
| **GLOBAL COGNITION** | | | | |  |
| **LE8 continuous** |  | **P-value** |  | **P-value** |  |
| LE8 score*Time | 0.0017 (0.0011; 0.0023) | <0.001 | 0.0013 (0.0007; 0.0018) | <0.001 | - |
| **LE8 categories** |  | **P for trend** |  | **P for trend** |  |
| Low score*Time | Reference |  | Reference |  | Reference |
| Moderate score*Time | 0.0036 (0.0013; 0.0060) | <0.001 | 0.0024 (0.0002; 0.0047) | 0.001 | 17% |
| High score*Time | 0.0073 (0.0042; 0.0105) |  | 0.0056 (0.0026; 0.0085) |  | 40% |

LE8 score: Life’s Essential 8 score divided by 10.

Low score: 0-49; Moderate score: 50-79; High score: 80-100

Model 1: linear mixed models with random intercepts and slopes adjusted for age at baseline, sex, race/ethnicity, education, and depression.

Inverse probability weighting for censoring was used to account for attrition bias.

^a^Difference between the slope of each category of the LE8 score and the slope of the first category divided by the slope of the first category multiplied by 100.

**Supplemental Table 4.** Association between baseline Life’s Essential 8’s diet categories and cognitive change over the study period.

|  | **Unadjusted** | | **Model 1** | |
| --- | --- | --- | --- | --- |
|  | **β (95% CI)** | **P for trend** | **β (95% CI)** | **P for trend** |
| **Memory** |  | |  | |
| Low score*Time | Reference | | Reference | |
| Moderate score*Time | 0.005 (0.002; 0.007) | <0.001 | 0.003 (0.000; 0.005) | 0.011 |
| High score*Time | 0.011 (0.002; 0.019) |  | 0.008 (0.000; 0.016) |  |
| **Verbal fluency** |  | |  | |
| Low score*Time | Reference |  | Reference |  |
| Moderate score*Time | 0.002 (0.000; 0.005) | 0.092 | 0.000 (-0.002; 0.002) | 0.985 |
| High score*Time | 0.004 (-0.004; 0.012) |  | 0.000 (-0.007; 0.008) |  |
| **Executive function** |  | |  | |
| Low score*Time | Reference | | Reference | |
| Moderate score*Time | 0.005 (0.002; 0.008) | 0.002 | 0.001 (-0.001; 0.004) | 0.355 |
| High score*Time | 0.006 (-0.004; 0.016) |  | 0.002 (-0.006; 0.011) |  |
| **Global cognition** |  | |  | |
| Low score*Time | Reference | | Reference | |
| Moderate score*Time | 0.003 (0.001; 0.005) | <0.001 | 0.001 (0.000; 0.003) | 0.133 |
| High score*Time | 0.006 (0.000; 0.012) |  | 0.004 (-0.002; 0.009) |  |

Low score: 0-49; Moderate score: 50-79; High score: 80-100

Model 1: linear mixed models with random intercepts and slopes adjusted for age at baseline, sex, race/ethnicity, education, and depression.

Inverse probability weighting for censoring was used to account for attrition bias.

**Supplemental Table 5.** Association between baseline Life’s Essential 8’s physical activity categories and cognitive change over the study period.

|  | **Unadjusted** | | **Model 1** | |
| --- | --- | --- | --- | --- |
|  | **β (95% CI)** | **P for trend** | **β (95% CI)** | **P for trend** |
| **Memory** |  | |  | |
| Low score*Time | Reference | | Reference | |
| Moderate score*Time | -0.003 (-0.010; 0.005) | 0.170 | -0.004 (-0.012; 0.002) | 0.393 |
| High score*Time | 0.002 (0.000; 0.005) |  | 0.000 (-0.003; 0.002) |  |
| **Verbal fluency** |  | |  | |
| Low score*Time | Reference | | Reference | |
| Moderate score*Time | 0.000 (-0.007; 0.008) | 0.093 | -0.001 (-0.008; 0.005) | 0.839 |
| High score*Time | 0.002 (0.000; 0.005) |  | 0.000 (-0.002; 0.003) |  |
| **Executive function** |  | |  | |
| Low score*Time | Reference | | Reference | |
| Moderate score*Time | 0.004 (-0.004; 0.014) | < 0.001 | 0.002 (-0.006; 0.010) | 0.033 |
| High score*Time | 0.007 (0.004; 0.010) |  | 0.003 (0.001; 0.006) |  |
| **Global cognition** |  | |  | |
| Low score*Time | Reference | | Reference | |
| Moderate score*Time | -0.001 (-0.007; 0.004) | 0.020 | -0.003 (-0.008; 0.002) | 0.378 |
| High score*Time | 0.002 (0.000; 0.004) |  | 0.000 (-0.001; 0.002) |  |

Low score: 0-49; Moderate score: 50-79; High score: 80-100

Model 1: linear mixed models with random intercepts and slopes adjusted for age at baseline, sex, race/ethnicity, education, and depression.

Inverse probability weighting for censoring was used to account for attrition bias.

**Supplemental Table 6.** Association between baseline Life’s Essential 8’s nicotine exposure categories and cognitive change over the study period.

|  | **Unadjusted** | | **Model 1** | |
| --- | --- | --- | --- | --- |
|  | **β (95% CI)** | **P for trend** | **β (95% CI)** | **P for trend** |
| **Memory** |  | |  | |
| Low score*Time | Reference | | Reference | |
| Moderate score*Time | -0.002 (-0.005; 0.002) | 0.612 | -0.002 (-0.006; 0.002) | 0.531 |
| High score*Time | -0.002 (-0.005; 0.002) |  | -0.002 (-0.005; 0.001) |  |
| **Verbal fluency** |  | |  | |
| Low score*Time | Reference | | Reference | |
| Moderate score*Time | 0.000 (-0.003; 0.004) | 0.263 | -0.001 (-0.005; 0.003) | 0.705 |
| High score*Time | -0.001 (-0.005; 0.002) |  | -0.001 (-0.005; 0.002) |  |
| **Executive function** |  | |  | |
| Low score*Time | Reference | | Reference | |
| Moderate score*Time | 0.004 (0.000; 0.009) | 0.075 | 0.001 (-0.002; 0.006) | 0.722 |
| High score*Time | 0.001 (-0.003; 0.005) |  | 0.001 (-0.002; 0.005) |  |
| **Global cognition** |  | |  | |
| Low score*Time | Reference | | Reference | |
| Moderate score*Time | -0.001 (-0.004; 0.002) | 0.216 | -0.001 (-0.004; 0.001) | 0.389 |
| High score*Time | -0.002 (-0.004; 0.000) |  | -0.001 (-0.004; 0.001) |  |

Low score: 0-49; Moderate score: 50-79; High score: 80-100

Model 1: linear mixed models with random intercepts and slopes adjusted for age at baseline, sex, race/ethnicity, education, and depression.

Inverse probability weighting for censoring was used to account for attrition bias.

**Supplemental Table 7.** Association between baseline Life’s Essential 8’s sleep health categories and cognitive change over the study period.

|  | **Unadjusted** | | **Model 1** | |
| --- | --- | --- | --- | --- |
|  | **β (95% CI)** | **P for trend** | **β (95% CI)** | **P for trend** |
| **Memory** |  | |  | |
| Low score*Time | Reference | | Reference | |
| Moderate score*Time | 0.000 (-0.007; 0.005) | 0.940 | -0.001 (-0.006; 0.005) | 0.943 |
| High score*Time | 0.000 (-0.005; 0.005) |  | -0.001 (-0.005; 0.004) |  |
| **Verbal fluency** |  |  |  |  |
| Low score*Time | Reference | | Reference | |
| Moderate score*Time | 0.001 (-0.005; 0.007) | 0.521 | 0.001 (-0.004; 0.006) | 0.832 |
| High score*Time | 0.002 (-0.003; 0.007) |  | 0.001 (-0.003; 0.006) |  |
| **Executive function** |  | |  | |
| Low score*Time | Reference | | Reference | |
| Moderate score*Time | 0.007 (0.000; 0.014) | 0.084 | 0.005 (-0.001; 0.011) | 0.248 |
| High score*Time | 0.007 (0.001; 0.013) |  | 0.004 (-0.001; 0.010) |  |
| **Global cognition** |  | |  | |
| Low score*Time | Reference | | Reference | |
| Moderate score*Time | 0.000(-0.004; 0.004) | 0.607 | 0.000 (-0.004; 0.004) | 0.934 |
| High score*Time | 0.001 (-0.002; 0.005) |  | 0.000 (-0.003; 0.004) |  |

Low score: 0-49; Moderate score: 50-79; High score: 80-100

Model 1: linear mixed models with random intercepts and slopes adjusted for age at baseline, sex, race/ethnicity, education, and depression.

Inverse probability weighting for censoring was used to account for attrition bias.

**Supplemental Table 8.** Association between baseline Life’s Essential 8’s body mass index categories and cognitive change over the study period.

|  | **Unadjusted** | | **Model 1** | |
| --- | --- | --- | --- | --- |
|  | **β (95% CI)** | **P for trend** | **β (95% CI)** | **P for trend** |
| **Memory** |  | |  | |
| Low score*Time | Reference | | Reference | |
| Moderate score*Time | 0.002 (0.000; 0.006) | 0.002 | 0.002 (0.000; 0.005) | 0.014 |
| High score*Time | -0.002 (-0.005; 0.001) |  | -0.001 (-0.004; 0.001) |  |
| **Verbal fluency** |  | |  | |
| Low score*Time | Reference | | Reference | |
| Moderate score*Time | 0.002 (-0.001; 0.005) | 0.318 | 0.002 (-0.001; 0.004) | 0.427 |
| High score*Time | 0.001 (-0.002; 0.004) |  | 0.001 (-0.001; 0.004) |  |
| **Executive function** |  | |  | |
| Low score*Time | Reference | | Reference | |
| Moderate score*Time | 0.002 (-0.001; 0.006) | 0.131 | 0.001 (-0.002; 0.005) | 0.074 |
| High score*Time | 0.004 (0.000; 0.007) |  | 0.003 (0.000; 0.007) |  |
| **Global cognition** |  | |  | |
| Low score*Time | Reference | | Reference | |
| Moderate score*Time | 0.002 (0.000; 0.005) | 0.015 | 0.002 (0.000; 0.004) | 0.050 |
| High score*Time | 0.000 (-0.002; 0.002) |  | 0.000 (-0.002; 0.002) |  |

Low score: 0-49; Moderate score: 50-79; High score: 80-100

Model 1: linear mixed models with random intercepts and slopes adjusted for age at baseline, sex, race/ethnicity, education, and depression.

Inverse probability weighting for censoring was used to account for attrition bias.

**Supplemental Table 9.** Association between baseline Life’s Essential 8’s blood lipid categories and cognitive change over the study period.

|  | **Unadjusted** | | **Model 1** | |
| --- | --- | --- | --- | --- |
|  | **β (95% CI)** | **P for trend** | **β (95% CI)** | **P for trend** |
| **Memory** |  | |  | |
| Low score*Time | Reference | | Reference | |
| Moderate score*Time | -0.001 (-0.003; 0.002) | 0.141 | 0.001 (-0.001; 0.004) | 0.467 |
| High score*Time | -0.003 (-0.006; 0.000) |  | 0.000 (-0.002; 0.003) |  |
| **Verbal fluency** |  | |  | |
| Low score*Time | Reference | | Reference | |
| Moderate score*Time | 0.000 (-0.002; 0.003) | 0.248 | 0.001 (-0.002; 0.003) | 0.506 |
| High score*Time | -0.002 (-0.005; 0.000) |  | -0.001 (-0.003; 0.001) |  |
| **Executive function** |  | |  | |
| Low score*Time | Reference | | Reference | |
| Moderate score*Time | -0.001 (-0.004; 0.002) | 0.774 | 0.000(-0.003; 0.003) | 0.572 |
| High score*Time | 0.000 (-0.003; 0.003) |  | 0.001 (-0.001; 0.004) |  |
| **Global cognition** |  | |  | |
| Low score*Time | Reference | | Reference | |
| Moderate score*Time | 0.000 (-0.002; 0.001) | 0.206 | 0.000 (-0.001; 0.002) | 0.841 |
| High score*Time | -0.002 (-0.004; 0.000) |  | 0.000 (-0.002; 0.002) |  |

Low score: 0-49; Moderate score: 50-79; High score: 80-100

Model 1: linear mixed models with random intercepts and slopes adjusted for age at baseline, sex, race/ethnicity, education, and depression.

Inverse probability weighting for censoring was used to account for attrition bias.

**Supplemental Table 10.** Association between baseline Life’s Essential 8’s blood glucose categories and cognitive change over the study period.

|  | **Unadjusted** | | **Model 1** | |
| --- | --- | --- | --- | --- |
|  | **β (95% CI)** | **P for trend** | **β (95% CI)** | **P for trend** |
| **Memory** |  | |  | |
| Low score*Time | Reference | | Reference | |
| Moderate score*Time | 0.010 (0.006; 0.014) | <0.001 | 0.010 (0.006; 0.014) | <0.001 |
| High score*Time | 0.005 (0.002; 0.008) |  | 0.006 (0.003; 0.009) |  |
| **Verbal fluency** |  | |  | |
| Low score*Time | Reference | | Reference | |
| Moderate score*Time | 0.002 (-0.002; 0.006) | 0.005 | 0.003 (0.000; 0.007) | <0.001 |
| High score*Time | 0.005 (0.002; 0.008) |  | 0.006 (0.003; 0.009) |  |
| **Executive function** |  | |  | |
| Low score*Time | Reference | | Reference | |
| Moderate score*Time | 0.006 (0.001; 0.011) | <0.001 | 0.006 (0.002; 0.011) | <0.001 |
| High score*Time | 0.008 (0.004; 0.011) |  | 0.009 (0.005; 0.012) |  |
| **Global cognition** |  | |  | |
| Low score*Time | Reference | | Reference | |
| Moderate score*Time | 0.006 (0.003; 0.009) | <0.001 | 0.007 (0.004; 0.009) | < 0.001 |
| High score*Time | 0.005 (0.003; 0.007) |  | 0.006 (0.004; 0.008) |  |

Low score: 0-49; Moderate score: 50-79; High score: 80-100

Model 1: linear mixed models with random intercepts and slopes adjusted for age at baseline, sex, race/ethnicity, education, and depression.

Inverse probability weighting for censoring was used to account for attrition bias.

**Supplemental Table 11.** Association between baseline Life’s Essential 8’s blood pressure categories and cognitive change over the study period.

|  | **Unadjusted** | | **Model 1** | |
| --- | --- | --- | --- | --- |
|  | **β (95% CI)** | **P for trend** | **β (95% CI)** | **P for trend** |
| **Memory** |  | |  | |
| Low score*Time | Reference | | Reference | |
| Moderate score*Time | 0.004 (0.000; 0.007) | <0.001 | 0.002 (0.000; 0.005) | < 0.001 |
| High score*Time | 0.007 (0.004; 0.010) |  | 0.008 (0.005; 0.010) |  |
| **Verbal fluency** |  | |  | |
| Low score*Time | Reference | | Reference | |
| Moderate score*Time | 0.005 (0.002; 0.008) | 0.002 | 0.004 (0.001; 0.007) | 0.005 |
| High score*Time | 0.005 (0.002; 0.008) |  | 0.004 (0.001; 0.007) |  |
| **Executive function** |  | |  | |
| Low score*Time | Reference | | Reference | |
| Moderate score*Time | 0.007 (0.003; 0.011) | <0.001 | 0.006 (0.002; 0.010) | <0.001 |
| High score*Time | 0.012 (0.009; 0.016) |  | 0.011 (0.008; 0.014) |  |
| **Global cognition** |  | |  | |
| Low score*Time | Reference | | Reference | |
| Moderate score*Time | 0.005 (0.002; 0.007) | <0.001 | 0.004 (0.001; 0.006) | <0.001 |
| High score*Time | 0.007 (0.005; 0.009) |  | 0.007 (0.005; 0.009) |  |

Low score: 0-49; Moderate score: 50-79; High score: 80-100

Model 1: linear mixed models with random intercepts and slopes adjusted for age at baseline, sex, race/ethnicity, education, and depression.

Inverse probability weighting for censoring was used to account for attrition bias.

**Supplemental Table 12.** Association between baseline Life’s Essential 8’s categories and global cognitive change over the study period stratified by age, sex, and race.

|  | **< 60 years old**  (n=9,244) | | **60 years old**  (n=2,146) | |
| --- | --- | --- | --- | --- |
|  | **β (95% CI)** | **P for trend** | **β (95% CI)** | **P for trend** |
| **Global cognition** |  | |  | |
| Low score*Time | Reference | | Reference | |
| Moderate score*Time | 0.0017 (-0.0011; 0.0046) | 0.113 | 0.0015 (-0.0057; 0.0088) | 0.257 |
| High score*Time | 0.0039 (0.0002; 0.0077) |  | 0.0088 (-0.0022; 0.0198) |  |
|  | **Female participants**  (n=6,334) | | **Male participants**  (n=5,056) | |
|  | **β (95% CI)** | **P for trend** | **β (95% CI)** | **P for trend** |
| **Global cognition** |  | |  | |
| Low score*Time | Reference | | Reference | |
| Moderate score*Time | 0.0006 (-0.0027; 0.0039) | 0.154 | 0.0030 (0.0001; 0.0068) | 0.015 |
| High score*Time | 0.0030 (-0.0007; 0.0076) |  | 0.0070 (0.0022; 0.0119) |  |
|  | **Black participants**  (n=4,916) | | **White participants**  (n=6,092) | |
|  | **β (95% CI)** | **P for trend** | **β (95% CI)** | **P for trend** |
| **Global cognition** |  | |  | |
| Low score*Time | Reference | | Reference | |
| Moderate score*Time | 0.0020 (-0.0012; 0.0058) | 0.117 | 0.0016 (-0.0016; 0.0050) | 0.106 |
| High score*Time | 0.0059 (0.0003; 0.0115) |  | 0.0041 (0.0001; 0.0083) |  |

Low score: 0-49; Moderate score: 50-79; High score: 80-100

Model 1: linear mixed models with random intercepts and slopes adjusted for age at baseline, sex, race/ethnicity, education, and depression.

Inverse probability weighting for censoring was used to account for attrition bias.

**Supplemental References**

[1. Molina M del CB, Faria CP de, Cardoso L de O, et al. Diet assessment in the Brazilian Longitudinal Study of Adult Health (ELSA-Brasil): Development of a food frequency questionnaire. *Rev Nutr*. 2013;26(2):167-176. doi:10.1590/S1415-52732013000200005](https://www.zotero.org/google-docs/?IRlQRG)

[2. Aquino EML, Barreto SM, Bensenor IM, et al. Brazilian Longitudinal Study of Adult Health (ELSA-Brasil): Objectives and Design. *Am J Epidemiol*. 2012;175(4):315-324. doi:10.1093/aje/kwr294](https://www.zotero.org/google-docs/?IRlQRG)

[3. Buuren S van, Groothuis-Oudshoorn K. **mice** : Multivariate Imputation by Chained Equations in *R*. *J Stat Softw*. 2011;45(3). doi:10.18637/jss.v045.i03](https://www.zotero.org/google-docs/?IRlQRG)

[4. Passos VM de A, Caramelli P, Benseñor I, Giatti L, Barreto SM. Methods of cognitive function investigation in the Longitudinal Study on Adult Health (ELSA-Brasil). *Sao Paulo Med J*. 2014;132(3):170-177. doi:10.1590/1516-3180.2014.1323646](https://www.zotero.org/google-docs/?IRlQRG)

[5. Bertola L, Benseñor IM, Barreto SM, et al. Measurement invariance of neuropsychological tests across different sociodemographic backgrounds in the Brazilian Longitudinal Study of Adult Health (ELSA-Brasil). *Neuropsychology*. 2020;34(2):227-234. doi:10.1037/neu0000597](https://www.zotero.org/google-docs/?IRlQRG)

[6. de Menezes ST, Giatti L, Brant LCC, et al. Hypertension, Prehypertension, and Hypertension Control: Association With Decline in Cognitive Performance in the ELSA-Brasil Cohort. *Hypertension*. 2021;77(2):672-681. doi:10.1161/HYPERTENSIONAHA.120.16080](https://www.zotero.org/google-docs/?IRlQRG)
